# The correlation of long non‐coding RNA intersectin 1‐2 with disease risk, disease severity, inflammation, and prognosis of acute ischemic stroke

**DOI:** 10.1002/jcla.23053

**Published:** 2019-10-24

**Authors:** Yi Zhang, Chenglin Niu

**Affiliations:** ^1^ Department of Neurology Shanxi Provincial People's Hospital Taiyuan China; ^2^ Department of ICU The Affiliated Brain Hospital of Nanjing Medical University Nanjing China

**Keywords:** acute ischemic stroke, disease severity, inflammation, long non‐coding RNA intersectin 1‐2, recurrence‐free survival

## Abstract

**Background:**

This study aimed to evaluate the predictive value of long non‐coding RNA intersectin 1‐2 (lnc‐ITSN1‐2) for acute ischemic stroke (AIS) risk, and investigate its correlation with disease severity, inflammation, and recurrence‐free survival (RFS) in AIS patients.

**Methods:**

Three hundred and twenty AIS patients were recruited, and plasma samples were collected within 24 hours after admission. lnc‐ITSN1‐2 expression form plasma was detected by reverse transcription‐quantitative polymerase chain reaction (RT‐qPCR). The National Institute of Health Stroke Scale (NIHSS) score was assessed, and RFS was calculated. Meanwhile, 320 controls were enrolled and plasma samples were collected on the enrollment, and lnc‐ITSN1‐2 expression was detected by RT‐qPCR.

**Results:**

lnc‐ITSN1‐2 expression was increased in AIS patients compared to controls (*P* < .001), and receiver operating characteristic curve revealed its predictive value for AIS risk (area under the curve: 0.804, 95% confidence interval, 0.763‐0.845). In AIS patients, lnc‐ITSN1‐2 expression was positively correlated with NIHSS score (*r* = 0.464, *P* < .001). For inflammation, lnc‐ITSN1‐2 expression was positively correlated with CRP (*r* = 0.398, *P* < .001), TNF‐α (*r* = 0.502, *P* < .001), IL‐1β (*r* = 0.313, *P* < .001), IL‐6 (*r* = 0.207, *P* < .001), IL‐8 (*r* = 0.400, *P* < .001), IL‐17 (*r* = 0.272, *P* < .001), and IL‐22 (*r* = 0.222, *P* < .001). In terms of predicted target microRNAs, lnc‐ITSN1‐2 expression was negatively correlated with microRNA (miR)‐107 (*r* = −0.467, *P* < .001), miR‐125a (*r* = −0.494, *P* < .001), and miR‐146a (*r* = −0.126, *P* = .025). For prognosis, high lnc‐ITSN1‐2 expression was correlated with worse RFS in AIS patients.

**Conclusion:**

lnc‐ITSN1‐2 exerts a good predictive value for AIS risk; meanwhile, its increased expression is correlated with enhanced disease severity, elevated inflammation, and worse RFS in AIS patients.

## 1 INTRODUCTION

Stroke, ranking as the second cause of worldwide mortality, influences over 17 million people and causes more than $300 billion in economic losses annually, which is divided into ischemic stroke (counting on over 80% of stroke incidences) and hemorrhagic stroke.^1^, ^2^, ^3^ Acute ischemic stroke (AIS), one of the common types of ischemic stroke, is caused by a deficiency of blood and oxygen supply to the brain tissue, subsequently leading to irreversible damage to the brain, and finally disability or even premature death within hours.^4^, ^5^ In such pathological processes of AIS, inflammation plays an important role by increasing neurocyte death and subsequently exacerbates the severity of AIS.^6^ Although current treatments against AIS (including intra‐arterial therapy and intravenous thrombolysis) have greatly progressed, there is still a part of patients who are unable to receive recommended therapy partly due to the narrow therapeutic window, causing over 3 million cases of mortality in 2017.^7^, ^8^, ^9^ Thus, it is necessary to search for new predictive biomarkers for early prevention and monitoring disease progression to improve prognosis in AIS patients.

Long non‐coding RNAs (lncRNAs), defined as non–protein‐coding RNAs with lengths exceeding 200 nucleotides, display various biological functions including chromatin modification, transcriptional regulation, post‐transcriptional regulation.^10^ Long non‐coding RNA intersectin 1‐2 (lnc‐ITSN1‐2) is a lncRNA located on chromatin 21 with a length of 451 bp and with NONCODE gene ID NONHSAG032726.2.^11^ The function of lnc‐ITSN1‐2 is reported by only a few studies, which reveal that it acts as a potential biomarker in inflammation‐related diseases (such as rheumatoid arthritis (RA), sepsis, and coronary artery disease (CAD)).^11^, ^12^, ^13^ Considering the abovementioned data and the implication of inflammation in AIS, we hypothesized that lnc‐ITSN1‐2 can also promote the pathological progression in AIS patients, while relevant research on the role of lnc‐ITSN1‐2 in AIS has not been studied before.^14^ Thus, we performed this study to explore the predictive value of lnc‐ITSN1‐2 for AIS risk and investigate its correlation with disease severity, inflammation, and recurrence‐free survival (RFS) in AIS patients.

## 2 MATERIALS AND METHODS

### 2.1 Patients

Between January 2013 and June 2016, 320 first‐episode AIS patients were consecutively enrolled in our hospital. The inclusion criteria were as follows: (a) newly diagnosed as AIS according to the criteria of World Health Organization (WHO),^15^ and confirmed by computed tomography (CT) scan, magnetic resonance imaging (MRI), and/or diffusion‐weighted imaging; (b) admitted to the hospital within 24 hours after the onset of symptoms; (c) no obvious abnormality in renal and hepatic functions; and (d) age ≥18 years. Patients were excluded in the following conditions: (a) presenting lacunar infarction or cerebral hemorrhagic infarction; (b) complicated with hematological malignancies or solid tumors; (c) severe infections, and inflammatory or autoimmune diseases; (d) died within 24 hours; (e) treatment with anti‐inflammatory drugs or immunosuppressive drugs within 3 months before enrollment; and (f) pregnant or lactating woman. In addition, 320 non‐AIS subjects who were complicated with stroke risk factors were recruited as controls. The screening criteria of controls included (a) complicated with at least three of following risk factors: hypertension, diabetes mellitus, heart disease, transient ischemic attack, obesity, hyperlipidemia, smoking, alcoholism, infections, platelet hyperaggregability, elevated blood lipid levels, and so on^15^; (b) no history of stroke, hematological malignancies, or solid tumors; (c) no severe infections, and inflammatory or autoimmune diseases; (d) age ≥18 years; and (e) not pregnant or lactating woman. This study was approved by the ethics committee of our hospital. All participants or their guardians provided written informed consents before enrollment.

### 2.2 Data collection

For all the participants, the clinical characteristics (age, gender, body mass index [BMI], current smoke, hypertension, hyperlipidemia, hyperuricemia, diabetes mellitus, and chronic kidney disease [CKD]) were recorded after the written informed consents were provided. Besides, C‐reactive protein (CRP) level was collected and the National Institute of Health Stroke Scale (NIHSS) score was assessed in the AIS patients. The NIHSS included 11 items (total score ranges from 0 to 42), and the higher score was corresponding to increased severity of stroke.^16^

### 2.3 Sample collection

Within 24 hours after admission, peripheral blood samples were collected from AIS patients, which were subsequently centrifuged at 1000 *g* for 20 minutes under 4°C. The plasma was separated and stored at −80°C for further detection. In addition, peripheral blood samples were also collected from controls on the enrollment, and the plasma was isolated using the same method described above.

### 2.4 lnc‐ITSN1‐2 and microRNA (miRNA) relative expression detection

The relative expression of lnc‐ITSN1‐2 in plasma of AIS patients and controls, and the relative expressions of microRNA (miR)‐107, miR‐125a, and miR‐146a in plasma of AIS patients were detected by reverse transcription‐quantitative polymerase chain reaction (RT‐qPCR). GAPDH was set as the internal reference for lnc‐ITSN1‐2, and U6 was set as the internal reference for miRNAs. RNA was extracted using a QIAamp RNA Blood Mini Kit (Qiagen) according to the manufacturer's instruction. Reverse transcription was performed using an iScript cDNA Synthesis Kit (Bio‐Rad). Polymerase chain reaction (PCR) was performed by THUNDERBIRD SYBR qPCR Mix (Toyobo). The quantitation of gene expression was calculated by the 2^‐ΔΔ^ *^c^* ^t^ method. The primer sequences are as follows: lnc‐ITSN1‐2, forward primer: GCTTCACTCGCTTGCTTACA, reverse primer: GGTTCTGTCTTGCCTTCTGTT; miR‐107, forward primer: ACACTCCAGCTGGGAGCAGCATTGTACAGG, reverse primer: TGTCGTGGAGTCGGCAATTC; miR‐125a, forward primer: ACACTCCAGCTGGGTCCCTGAGACCCTTTA, reverse primer: TGTCGTGGAGTCGGCAATTC; miR‐146a, forward primer: ACACTCCAGCTGGGTGAGAACTGAATTCCA, reverse primer: TGTCGTGGAGTCGGCAATTC; GAPDH, forward primer: TGACCACAGTCCATGCCATCAC reverse primer: GCCTGCTTCACCACCTTCTTGA; U6, forward primer: CTCGCTTCGGCAGCACATATACTA, reverse primer: ACGAATTTGCGTGTCATCCTTGC.

### 2.5 Enzyme‐linked immune sorbent assay

The levels of inflammatory cytokines in plasma of AIS patients, including tumor necrosis factor‐α (TNF‐α), interleukin‐1β (IL‐1β), IL‐6, IL‐8, IL‐17, and IL‐22 were measured using commercial human enzyme‐linked immune sorbent assay (ELISA) kits (Abcam) following the manufacturer's protocol.

### 2.6 Follow‐up

After enrollment, all AIS patients received routine treatments based on their clinical status. Regular follow‐up was conducted for the AIS patients until 36 months or stroke recurrence or death, and the median follow‐up duration was 36 months (range 0.0‐36.0 months). During follow‐up, stroke recurrence or death was recorded, and RFS was calculated from the date of admission to the date of stroke recurrence or death. Besides, 38 (11.9%) AIS patients lost follow‐up, and in the final analysis, they were censored on the date of stroke recurrence or last visit.

### 2.7 Statistical analysis

Continuous variables were expressed as mean ± standard deviation (SD) or median (interquartile range, IQR), while categorical variables were expressed as count (percentage). Comparison between two groups was determined by Student's *t* test, the chi‐square test, or the Wilcoxon rank‐sum test. Correlation between continuous variables was analyzed by Spearman's rank correlation test. Receiver operating characteristic (ROC) curve and the area under the curve (AUC) with 95% confidence interval (CI) were used to assess the ability of lnc‐ITSN1‐2 in discriminating AIS patients and controls. RFS was displayed by the Kaplan‐Meier curve, and the difference in RFS between two groups was determined by log‐rank test. SPSS 24.0 statistical software (IBM) was used for statistical analysis, and GraphPad Prism 7.00 software (GraphPad Software) was used for figures plotting. *P* value <.05 was considered significant.

## 3 RESULTS

### 3.1 Baseline characteristics

For demographic characteristics, the mean age in AIS patients and controls was 62.6 ± 10.8 years and 61.6 ± 9.1 years, respectively. There were 76 (23.8%) females and 244 (76.3%) males in AIS patients, and 65 (20.3%) females and 255 (79.7%) males in the controls. The mean value of BMI in AIS patients and the controls was 24.7 ± 2.9 kg/m^2^ and 24.2 ± 2.8 kg/m^2^, respectively. Compared with the controls, BMI was higher in AIS patients (*P* = .013), while there was no difference in age (*P* = .225) and gender (*P* = .294) between AIS patients and controls. For clinical characteristics, the numbers of patients with hypertension, hyperlipidemia, hyperuricemia, diabetes mellitus, and CKD were 274 (85.6%), 156 (48.8%), 107 (33.4%), 77 (24.1%), and 51 (15.9%), respectively, in AIS patients, while those were 239 (74.7%), 150 (46.9%), 101 (31.6%), 60 (18.8%), and 30 (9.4%), respectively, in controls. Compared with controls, the numbers of patients with hypertension (*P* = .001) and CKD (*P* = .013) were increased in AIS patients, whereas no difference was observed in occurrences of smoking behavior (*P* = .576), hyperlipidemia (*P* = .635), hyperuricemia (*P* = .613), or diabetes mellitus (*P* = .101) between AIS patients and the controls. Other baseline characteristics are presented in Table 1.

**Table 1:** Clinical characteristics of AIS patients and controls

Items	Controls (N = 320)	AIS patients (N = 320)	*P* value
Age (y), mean ± SD	61.6 ± 9.1	62.6 ± 10.8	.225
Gender, No. (%)			
Female	65 (20.3)	76 (23.8)	.294
Male	255 (79.7)	244 (76.3)	
BMI (kg/m^2^), mean ± SD	24.2 ± 2.8	24.7 ± 2.9	.013
Current smoke, No. (%)			
No	186 (58.1)	179 (55.9)	.576
Yes	134 (41.9)	141 (44.1)	
Common complications, No. (%)			
Hypertension	239 (74.7)	274 (85.6)	.001
Hyperlipidemia	150 (46.9)	156 (48.8)	.635
Hyperuricemia	101 (31.6)	107 (33.4)	.613
Diabetes mellitus	60 (18.8)	77 (24.1)	.101
CKD	30 (9.4)	51 (15.9)	.013
NIHSS score, mean ± SD	–	8.4 ± 3.5	–
Biochemical indexes, median (IQR)	–		–
CRP (mg/L)	–	41.5 (32.5‐57.2)	–
TNF‐α (pg/mL)	–	41.2 (29.5‐57.8)	–
IL‐1β (pg/mL)	–	89.8 (61.5‐148.4)	–
IL‐6 (pg/mL)	–	7.9 (4.9‐10.7)	–
IL‐8 (pg/mL)	–	71.0 (56.0‐93.1)	–
IL‐17 (pg/mL)	–	79.0 (56.2‐113.6)	–
IL‐22 (pg/mL)	–	148.9 (92.3‐201.7)	–

Note

Comparison was determined by Student's t test or the chi‐square test.

Abbreviations: AIS, acute ischemic stroke; BMI, body mass index; CKD, chronic kidney disease; CRP, C‐reactive protein; IL, interleukin; IQR, interquartile range; NIHSS, National Institute of Health stroke scale; SD, standard deviation; TNF‐α, tumor necrosis factor‐α.

### 3.2 The expression of lnc‐ITSN1‐2 and its predictive value for AIS risk

**Figure 1:**
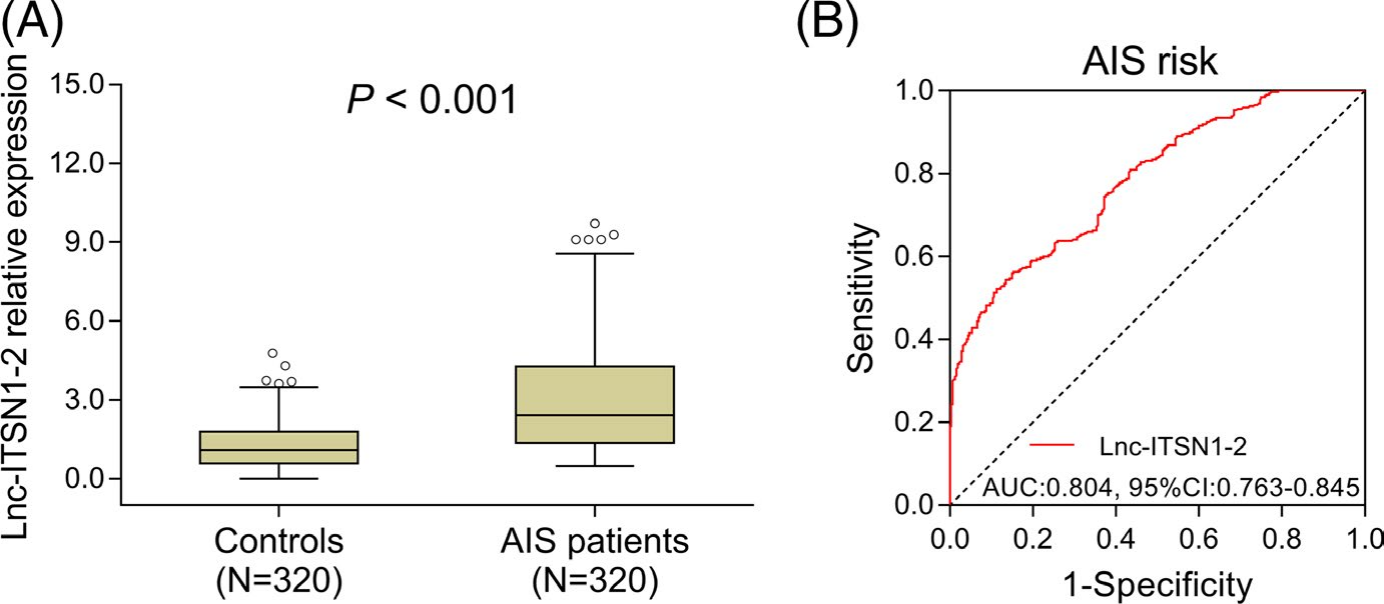
Comparison of lnc‐ITSN1‐2 expression between AIS patients and controls. A, Comparison of lnc‐ITSN1‐2 expression between AIS patients and the controls. B, Predictive value of lnc‐ITSN1‐2 for AIS risk. Comparison between groups was performed by the Wilcoxon rank‐sum test. ROC curve was conducted, and AUC was calculated to evaluate the predictive value of lnc‐ITSN1‐2 for AIS risk. *P* value <.05 was considered significant. lnc‐ITSN1‐2, long non‐coding RNA intersectin 1‐2; AIS, acute ischemic stroke; ROC, receiver operating characteristic; AUC, area under the curve; CI, confidence interval

The median value of lnc‐ITSN1‐2 expression was 2.421 (1.361‐4.274) in AIS patients and 1.098 (0.587‐1.798) in the controls, and its expression was increased in AIS patients compared with the controls (*P* < .001; Figure 1A**)**. In addition, the ROC curve revealed that lnc‐ITSN1‐2 presented with a good predictive value for increased AIS risk (AUC: 0.804, 95% CI: 0.763‐0.845; Figure 1B).

### 3.3 Correlation of lnc‐ITSN1‐2 expression with NIHSS score

**Figure 2:**
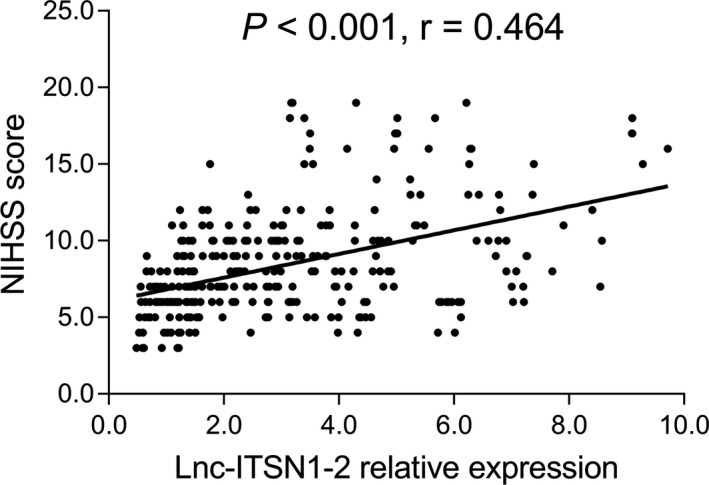
Association of lnc‐ITSN1‐2 expression with NIHSS score in AIS patients. Correlation of lnc‐ITSN1‐2 expression with NIHSS score was performed by Spearman's rank correlation test. *P* value <.05 was considered significant. lnc‐ITSN1‐2, long non‐coding RNA intersectin 1‐2; NIHSS, National Institute of Health Stroke Scale

In order to evaluate the potential of lnc‐ITSN1‐2 as a biomarker for monitoring disease severity in AIS patients, NIHSS score was assessed and the correlation between lnc‐ITSN1‐2 expression and NIHSS score was performed, which displayed that lnc‐ITSN1‐2 expression was positively associated with NIHSS score in AIS patients (*r* = 0.464, *P* < .001; Figure 2).

### 3.4 Association of lnc‐ITSN1‐2 expression with common complications

lnc‐ITSN1‐2 high expression was correlated with increased occurrence of hypertension (*P* = .013), while there was no correlation of lnc‐ITSN1‐2 expression with hyperlipidemia (*P* = .615), hyperuricemia (*P* = .191), diabetes mellitus (*P* = .307), or CKD (*P* = .169) in AIS patients (Table 2).

**Table 2:** Correlation of lnc‐ITSN1‐2 relative expression with common complications in AIS patients

Items	lnc‐ITSN1‐2, median (IQR)	*P* value
Hypertension		
No	1.234 (0.699‐2.771)	.013
Yes	1.618 (0.961‐2.840)	
Hyperlipidemia		
No	1.472 (0.929‐2.603)	.615
Yes	1.617 (0.860‐2.876)	
Hyperuricemia		
No	1.367 (0.973‐2.517)	.191
Yes	1.627 (0.899‐2.871)	
Diabetes mellitus		
No	1.497 (0.925‐2.191)	.307
Yes	1.579 (0.900‐2.922)	
CKD		
No	1.532 (0.904‐2.748)	.169
Yes	1.880 (0.952‐3.565)	

Note

Comparison was determined by the Wilcoxon rank‐sum test.

Abbreviations: AIS, acute ischemic stroke; CKD, chronic kidney disease; IQR, interquartile range.

### 3.5 Correlation of lnc‐ITSN1‐2 expression with inflammation

lnc‐ITSN1‐2 expression was positively associated with the levels of CRP (*r* = 0.398, *P* < .001), TNF‐α (*r* = 0.502, *P* < .001), IL‐1β (*r* = 0.313, *P* < .001), IL‐6 (*r* = 0.207, *P* < .001), IL‐8 (*r* = 0.400, *P* < .001), IL‐17 (*r* = 0.272, *P* < .001), and IL‐22 (*r* = 0.222, *P* < .001) in AIS patients (Table 3).

**Table 3:** Correlation of lnc‐ITSN1‐2 relative expression with inflammatory markers

Items		CRP	TNF‐α	IL‐1β	IL‐6	IL‐8	IL‐17	IL‐22
lnc‐ITSN1‐2	*P* value	<.001	<.001	<.001	<.001	<.001	<.001	<.001
	Correlation coefficient (*r*)	0.398	0.502	0.313	0.207	0.400	0.272	0.222

Note

Correlation was determined by Spearman's rank correlation test.

Abbreviations: CRP, C‐reactive protein; IL, interleukin; TNF‐α, tumor necrosis factor‐α.

### 3.6 Correlation of lnc‐ITSN1‐2 expression with RFS

**Figure 3:**
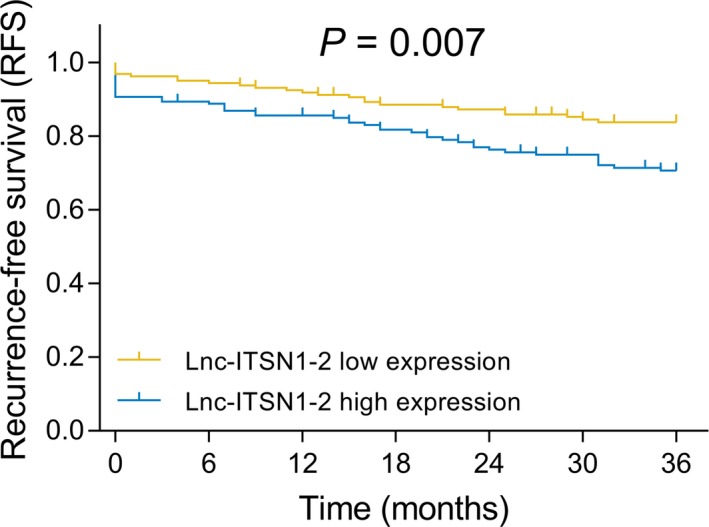
Association of lnc‐ITSN1‐2 expression with RFS in AIS patients. Kaplan‐Meier curve was conducted to display RFS. Comparison of RFS between the lnc‐ITSN1‐2 high‐expression group and the lnc‐ITSN1‐2 low‐expression group was conducted by log‐rank test. *P* value <.05 was considered significant. lnc‐ITSN1‐2, long non‐coding RNA intersectin 1‐2; RFS, recurrence‐free survival

According to the median value of lnc‐ITSN1‐2 expression in AIS patients, the patients were further divided into two groups: the lnc‐ITSN1‐2 high‐expression group and the lnc‐ITSN1‐2 low‐expression group, and the Kaplan‐Meier curve was performed to investigate the correlation between lnc‐ITSN1‐2 expression and RFS in AIS patients, which presented that RFS was poorer in the lnc‐ITSN1‐2 high‐expression group compared with the lnc‐ITSN1‐2 low‐expression group (*P* = .007; Figure 3).

### 3.7 Correlation of lnc‐ITSN1‐2 expression with predicted target miRNAs

**Figure 4:**
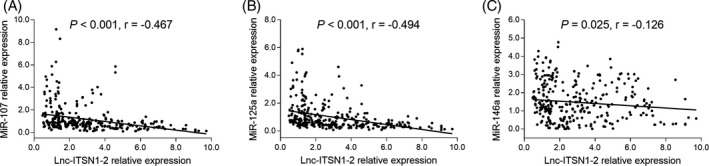
Association of lnc‐ITSN1‐2 expression with predicted target miRNAs in AIS patients. A, Correlation of lnc‐ITSN1‐2 expression with miR‐107. B, Correlation of lnc‐ITSN1‐2 expression with miR‐125a. C, Correlation of lnc‐ITSN1‐2 expression with miR‐146a. Correlations between lnc‐ITSN1‐2 expression and miRNAs expressions were determined by Spearman's rank correlation test. *P* value <.05 was considered significant. lnc‐ITSN1‐2, long non‐coding RNA intersectin 1‐2; miRNAs, microRNAs; miR‐107, microRNA‐107; miR‐125a, microRNA‐125a; miR‐146a, microRNA‐146a

Considering miR‐107, miR‐125a, and miR‐146a were predicted to be target genes of lnc‐ITSN1‐2 by starBase and miRcode database, and were well‐known inflammation‐related miRNAs, we further detected the expressions of these three miRNAs and discovered that lnc‐ITSN12 expression was negatively correlated with miR‐107 (*r* = −0.467, *P* < .001; Figure 4A), miR‐125a (*r* = −0.494, *P* < .001; Figure 4B), and miR‐146a (*r* = −0.126, *P* = .025; Figure 4C) expressions in AIS patients.

## 4 DISCUSSION

In the present study, we discovered that (a) lnc‐ITSN1‐2 was highly expressed in AIS patients compared to the controls, and it displayed a good predictive value for AIS risk; (b) lnc‐ITSN1‐2 high expression was associated with worse disease severity and increased inflammation in AIS patients; and (c) lnc‐ITSN1‐2 high expression was associated with poor RFS in AIS patients.

From pathological perspective of AIS, inflammation serves as a critical part in the disease exacerbation, which not only directly affects inflammatory pathways (including nuclear factor kappa‐light‐chain‐enhancer of activated B cell (NF‐κB) and Toll‐like receptor (TLR) pathways) to damage vascular wall, then changes vascular structure and promotes atherosclerosis, thereby decreasing blood flowing and increasing risk of AIS,^17^ but also indirectly increases neurocyte death through inducing TNF‐dependent apoptosis or necrosis in ischemic conditions, thereby accelerating the tissue damage in relevant part of the brain, retina, or spinal cord and facilitating the progression of AIS.^18^ According to the previous studies, several lncRNAs act as enhancers of inflammatory responses in AIS by activating inflammation‐related pathways such as NF‐κB pathway, TRL pathway, and JAK/STAT pathway.^19^, ^20^, ^21^, ^22^ For instance, increased lncRNA H19 expression is associated with impaired neurological function and increased TNF‐α level in AIS animal models.^23^ Antisense non‐coding RNA in the cyclin‐dependent kinase inhibitor 4 locus (ANRIL), an antisense lncRNA co‐clustered with p15/CDKN2B‐p16/CDKN2A‐p14/ARF, is overexpressed in cerebral infarction rat models and plays a pro‐inflammatory role by activating NF‐κB pathway.^24^ Likewise, lncRNA Gm4419 could activate NF‐κB pathway and contributes to cell damage in oxygen‐glucose–deprived cerebral microglial cells.^25^ Another in vitro and in vivo study discloses that lncRNA SNHG14 elevates the expression of pro‐inflammatory factors (such as TNF‐α and nitric oxide), thereby aggravating neuron damage by regulating miR‐145‐5p/PLA2G4A.^26^ Therefore, these previous findings suggest that lncRNAs might be regulators in inflammation or biomarkers for disease progression in AIS.

In view of lnc‐ITSN1‐2, although there are three previous studies elucidating the potential of lnc‐ITSN1‐2 for the disease risk of inflammation‐related diseases (including RA, sepsis, and CAD), no previous study has been carried out to explore the role of lnc‐ITSN1‐2 in AIS until now. Considering the predictive value of lnc‐ITSN1‐2 for the increased risk in these inflammation‐related diseases, and meanwhile the strong relationship of AIS with inflammation due to ischemic conditions, we hypothesized that lnc‐ITSN1‐2 could predict higher AIS risk as well. Therefore, we performed this study to detect the lnc‐ITSN1‐2 expression in AIS patients and its predictive value for AIS risk, and we discovered that lnc‐ITSN1‐2 expression was elevated in AIS patients compared with the controls, and it exerted a good predictive value for increased AIS risk. Possible explanations for these results might be that lnc‐ITSN1‐2 upregulated several inflammatory cytokines (including TNF‐α, IL‐6, and IL‐8) and inflammation‐related pathways (including NF‐κB and TRL pathways) to advocate inflammation, which subsequently increased the vascular damage and altered the vascular structure, thereby leading to elevated risk in ischemia, which resulted in the enhanced risk of AIS.

Similarly, few studies have been performed to investigate the role of lnc‐ITSN1‐2 in inflammation‐related diseases. Just three previous studies reveal that the enhanced lnc‐ITSN1‐2 expression is associated with elevated inflammation and disease severity of RA, CAD, and sepsis.^11^, ^12^, ^13^ For instance, a previous study reveals that lnc‐ITSN1‐2 is positively correlated with disease activity score in 28 joints, as well as CRP in RA patients.^11^ Another study discloses that lnc‐ITSN1‐2 is positively associated with acute physiology and chronic health evaluation II score, as well as inflammatory factor expressions (including CRP, TNF‐α, IL‐1β, IL‐6, IL‐8, IL‐10, and IL‐17) in sepsis patients.^12^ However, no research has been done to explore the correlation of lnc‐ITSN1‐2 with diseases severity and inflammation in AIS patients. In this study, positive correlation of lnc‐ITSN1‐2 expression with the NIHSS score and inflammatory markers levels (including CRP, TNF‐α, IL‐1β, IL‐6, IL‐8, IL‐17, and IL‐22) and negative correlation of lnc‐ITSN1‐2 expression with potential targeting miRNAs (including miR‐107, miR‐125a, and miR‐146a), which indicated that lnc‐ITSN1‐2 high expression was correlated with worse disease severity and increased inflammation in AIS patients, were discovered. These results could be explained by that (a) lnc‐ITSN1‐2 suppressed several gene expression and inhibited their anti‐inflammatory effect (including miR‐107, miR‐125a, and miR‐146a [abovementioned]) to cause the activation of pro‐inflammatory pathways (including NF‐κB pathway and TRL pathway), thereby upregulating these pro‐inflammatory markers (including CRP, TNF‐α, IL‐1β, and IL‐6), which eventually promoted inflammatory response and increased diseases severity in AIS patients. (b) lnc‐ITSN1‐2 inhibited the anti‐angiogenesis effect of miR‐107, miR‐125a, and miR‐146a to increase the alteration of vascular structure, thereby increasing disease severity in AIS patients.^27^, ^28^

In order to investigate the correlation of lnc‐ITSN1‐2 expression with AIS patients’ prognosis, we further recorded stroke recurrence and death with follow‐ups of 36 months, and we discovered that increased lnc‐ITSN1‐2 expression was associated with worse RFS in AIS patients. The possible reasons might be that: (a) lnc‐ITSN1‐2 increased inflammation to result in an enhanced severity of AIS, finally causing worse RFS in AIS patients (abovementioned); (b) lnc‐ITSN1‐2 might activate or suppress several pathways to induce drug resistance, reducing treatment effect and causing worse RFS in AIS patients, while the underlying molecular mechanism needed further exploration.

There were limitations existing in this study. (a) The follow‐up in this study was 36 months; hence, the long‐term influence of lnc‐ITSN1‐2 expression on the recovery in AIS patients was not conducted, which could be investigated further. (b) In order to avoid interference, only the first‐episode AIS patients were enrolled; thus, lnc‐ITSN1‐2 expression and its correlation with disease severity, inflammation, and RFS in patients with relapsed AIS should be studied further. (c) The specific mechanism of lnc‐ITSN1‐2 in the genesis and progression of AIS was not explored. (d) Patients who received thrombolysis were included in this study, which might be an extremely compounding factor. Due to that the administration of thrombolysis might decrease disease severity and improve prognosis in AIS patients, the effect of lnc‐ITSN1‐2 on disease severity, inflammation, and RFS in AIS patients might be influenced. (e) Due to that the AIS patients who died within 24 hours were with worse disease severity, which might cause deviation in this study, also for these patients who died within 24 hours, there might not be enough time to collect blood samples and clinical data. Hence, based on the above reasons, patients died within 24 hours were excluded in this study, which might be a bias, and therefore, a further study including them is needed. (f) As the expressions of targeted miRNAs in controls were not detected, a further study is needed.

In conclusion, lnc‐ITSN1‐2 displays a good predictive value for AIS risk, and it is correlated with increased disease severity and inflammation, as well as worse RFS in AIS patients, which provides a potential biotarget for early prevention and monitoring disease progression to further improve prognosis in AIS patients.
